# Protocol for conducting a systematic review on diagnostic accuracy in clinical research

**DOI:** 10.1016/j.mex.2024.102569

**Published:** 2024-01-20

**Authors:** Marco Sguanci, Stefano Mancin, Michela Piredda, Maria Grazia De Marinis

**Affiliations:** a Department of Medicine and Surgery, Research Unit of Nursing Science, Università Campus Bio-Medico di Roma, Roma, Italy; b Department of Biomedicine and Prevention, University of Rome “Tor Vergata”, Rome, Italy; c IRCCS Humanitas Research Hospital Rozzano, Milan, Italy; d Fondazione Policlinico Universitario Campus Bio-Medico, Roma, Italy

**Keywords:** Protocol, Screening tool, Diagnostic accuracy study, Systematic review, Systematic Review on diagnostic accuracy in clinical research

## Abstract

In the landscape of modern medicine, the ability to accurately diagnose various clinical conditions is paramount. As new diagnostic tools continue to emerge, their accuracy must be rigorously assessed before clinical implementation. This paper introduces a systematic review protocol tailored for diagnostic accuracy studies, drawing inspiration from a review on dysphagia screening in post-stroke patients. The protocol, designed with precision and transparency at its core, facilitates a thorough synthesis of evidence, employing tools such as the Quality Assessment of Diagnostic Accuracy Studies-2 (QUADAS-2) and the Standards for Reporting of Diagnostic Accuracy Studies (STARD) checklist for robust evaluation. The protocol emphasizes registration with the PROSPERO database and adherence to Preferred Reporting Items for Systematic Reviews and Meta-Analyses (PRISMA) guidelines. The systematic search approach encompasses a comprehensive exploration of databases and precise keyword combinations. Distinctive inclusion and exclusion criteria, coupled with a dual-reviewer methodology, ensure the selection of high-quality studies. This framework has the potential to serve as a benchmark for systematic reviews in diagnostic accuracy, highlighting the importance of standardization, transparency, and adaptability in clinical research. This approach paves the way for a research methodology that delves deeper into diagnostic tools across various clinical scenarios, promoting evidence-based advancements in patient care.

## Graphical abstract

**Figure:**
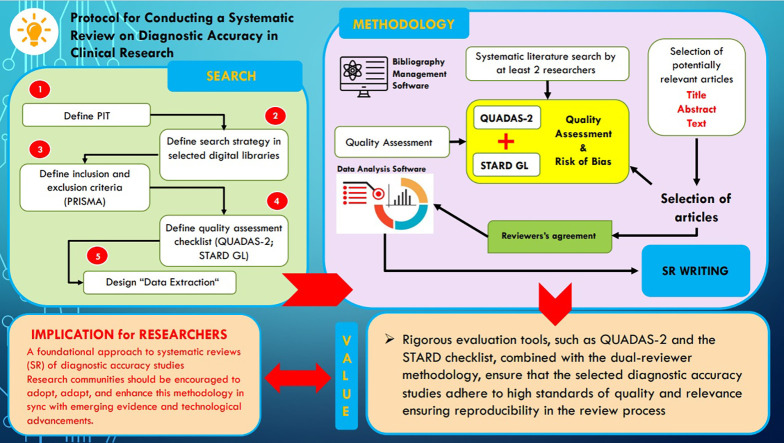

Specifications table

**Table:** 

Subject area:	Medicine and Dentistry
More specific subject area:	Evidence Based Medicine and clinical research
Name of your protocol:	Protocol for conducting Systematic Review of Dysphagia screening tools in chronic post-stroke patients
Reagents/tools:	IBM SPSS Statistics for Windows, version 23.0; IBM Corp
Experimental design:	Not Applicable
Trial registration:	The protocol of this systematic review was registered in the International prospective register of system- atic reviews (PROSPERO) of the National Institute of Health Research available at https://www.crd.york. ac.uk/prospero/ with protocol registration number: CRD42022372303.
Ethics:	Not Applicable
Value of the Protocol:	• *Rigor and Precision:* The protocol ensures a high standard of rigor in the selection and evaluation of studies, ensuring that every decision is based on a thorough assessment and is free from individual biases. The adoption of tools such as QUADAS-2 and the STARD Checklist reinforces the objectivity and quality of the review. • *Transparency and Reproducibility:* Each step of the protocol is outlined to guarantee maximum transparency, allowing other researchers to replicate and adapt the methodology in future studies. The accessibility of search strategies and inclusion/exclusion criteria ensures that the process can be clearly understood and followed by others. • *Versatility and Adaptability:* The adoption of standardized evaluation tools, combined with a detailed review methodology, ensures reproducibility in the review process; the described protocol can be used for all types of systematic reviews about diagnostic accuracy studies and related screening tools

## Background

Modern medicine largely hinges on the ability to diagnose a broad array of clinical conditions and diseases accurately and promptly. While new diagnostic tools and techniques are continually emerging, it's imperative to rigorously assess their accuracy before incorporating them into clinical practice [1]. This leads us to the realm of diagnostic accuracy studies, i.e., specific research designs aimed at gauging how well a diagnostic tool or procedure pinpoints a given condition compared to a defined reference standard [2].

Systematic reviews on diagnostic accuracy studies strive to synthesize and critically evaluate the existing literature on a particular diagnostic tool or procedure [3]. These reviews provide a holistic assessment of a diagnostic tool's performance, factoring in variables such as sensitivity, specificity, predictive values, and other related parameters. Such a review type gains significance given the plethora and diversity of available diagnostic tools and techniques and the need to fathom their strengths and limitations in varied clinical settings [4,5].

An exemplar of the importance of these studies can be traced to a recently published systematic review [6] on dysphagia screening in post-stroke patients. Even though it geared specifically towards dysphagia screening tools in chronic stroke patients, its methodological approach underscored the universal significance and the imperative for well-structured protocols in conducting systematic reviews of diagnostic accuracy.

### Research protocol objective

Drawing inspiration from the methodology employed in the aforementioned study, and recognizing the demand for a standardized approach in systematic reviews, this protocol seeks to deliver a clear and rigorous framework for conducting a systematic review in the landscape of diagnostic accuracy studies. This framework, while adaptable, aspires to uphold the requisite high caliber and integrity for assessing diagnostic tools across varied clinical scenarios. The protocol is tailored to navigate researchers through the intricacies of diagnostic accuracy studies, ensuring each step, from articulating the research query to synthesizing findings, is executed with precision and transparency.

## Methods

### Research design

To provide a thorough synthesis of existing evidence derived from a specific topic (e.g.: dysphagia screening tools in chronic post-stroke patients), we have structured a protocol for a systematic review of diagnostic accuracy studies as well as systematic reviews.

The methodology employed in this protocol highlights the importance of conducting thorough research with a strong emphasis on both methodological rigor and the relevance of the selected studies. Data extraction must be carried out consistently, followed by a comprehensive synthesis to provide a comprehensive overview.

### Protocol registration

Before drafting a systematic review, it is essential to register the protocol in the international PROSPERO database (https://www.crd.york.ac.uk/prospero/), which is managed by the National Institute of Health Research. This registration serves to guarantee that the review is carried out with the highest levels of transparency and integrity.

### Review methodology and definition of the research question

The review must be conducted and reported according to the Preferred Reporting Items for a Systematic Review and Meta-analysis of Diagnostic Test Accuracy Studies Guidelines [7].

Adopting PRISMA will ensure that the systematic review is performed and reported with precision and transparency. This initial step is crucial for gaining an in-depth understanding of current practices and official recommendations in the study field. Using PRISMA guidelines, one can also outline the inclusion and exclusion criteria.

The definition of the research question should be conducted in accordance with the Cochrane Manual for Systematic Reviews of the Accuracy of Diagnostic Tests, [8] an official guide detailing the process of preparing and maintaining systematic reviews of the accuracy of diagnostic tests; in order to process this study question, the PIT (population, index test(s), target condition) methodology should be used [8] (Supplementary file 1).

### Search methods for the identification of studies

Initially, relevant guidelines from leading scientific societies should be identified. Upon this foundation, a systematic and comprehensive search should be conducted across databases such as Cochrane Library, Pubmed, Embase, Cinahl, Scopus, and Web of Science. To further enhance this search, manual review of reference lists from relevant articles is recommended, coupled with the use of academic search engines like Google Scholar to pinpoint additional publications from the grey literature. The search strategy should involve the utilization of standardized terms, supplemented with keywords germane to the subject of interest. These keywords should be judiciously combined using Boolean operators, specifically "AND" and "OR", in search strings tailored according to the distinct characteristics of each database. The scope of the search should encompass literature published up to a specified date. Detailed search algorithms would be available for reference through a provided data availability statement. To ensure objectivity and minimize bias in such a specialized study, it is recommended that at least two academic researchers collaboratively undertake the search. This cooperative methodology would facilitate the juxtaposition and contrast of search outcomes, thereby amplifying the reliability of the article selection procedure.

Upon the completion of the database search, the retrieved records should be exported to bibliographic management software, such as EndNote (©2023 Clarivate). Utilizing the software's inherent automatic duplicate detection features is advised to identify and eliminate duplicated records. Additionally, a manual review should be instituted to further ascertain the omission of any residual duplicates, thereby guaranteeing a pristine and duplicate-free record set. Subsequently, titles and abstracts of the recognized articles should be meticulously assessed to pinpoint potentially pertinent publications. To ascertain eligibility, an exhaustive examination of the full texts of these chosen articles is warranted. It's paramount to emphasize that the entire identification and selection process should be executed independently by a minimum of two researchers. In instances where discrepancies emerge, involving a third reviewer to reach a consensus decision is prudent.

**Fig. 1:**
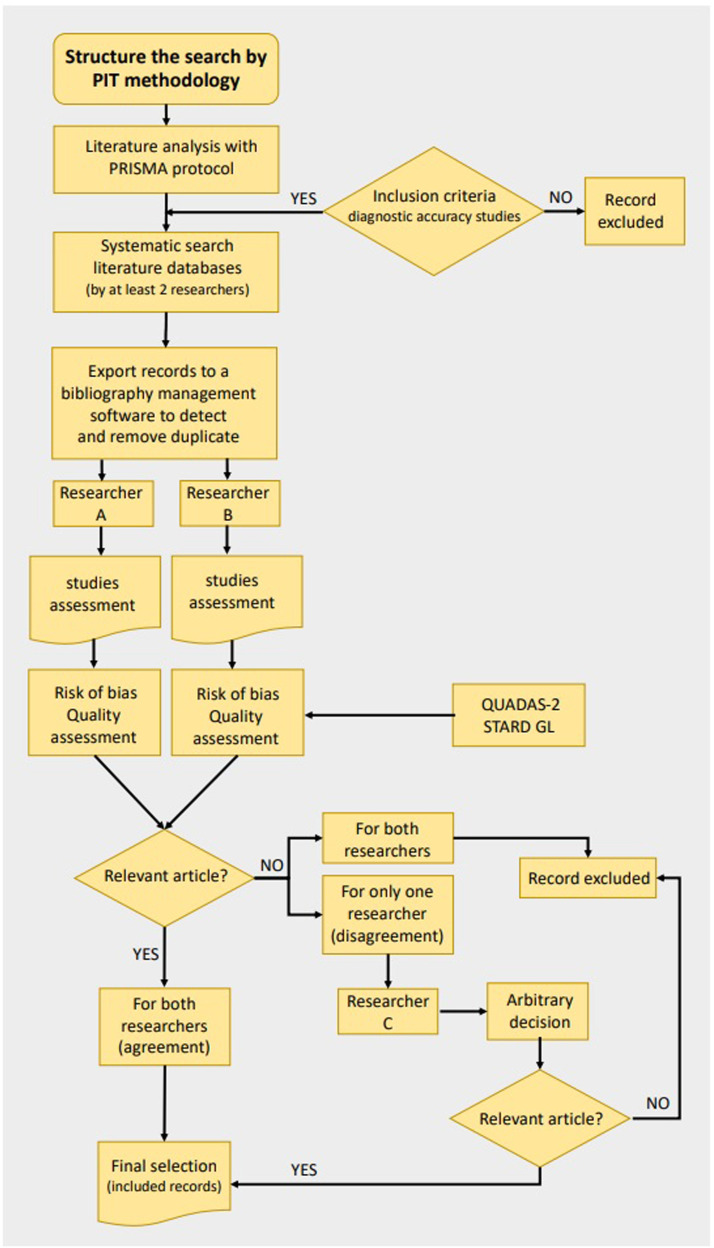
Search strategy methodology. PIT: patient/population, index test(s), target condition; PRISMA: Preferred Reporting Items for a Systematic Review and Meta-analysis of Diagnostic Test Accuracy Studies Guidelines; QUADAS-2: Quality Assessment of Diagnostic Accuracy Studies; STARD GL: Standards for Reporting of Diagnostic Accuracy Studies).

Lastly, for the sake of transparency and reproducibility, all search strategies employed in the study, including specific search strings, should be meticulously recorded. This documentation should be made available in a supplementary document (Supplementary File 1) accompanying the research findings (Fig. 1).

### Inclusion and exclusion criteria

Given the nature of publications concerning diagnostic accuracy studies, adopting a methodical approach would aim to ensure the consideration of only top-tier and pertinent sources, thus sidestepping potential biases from materials potentially lacking rigorous quality standards.

For clarity in a typical review, the inclusion criteria might be delineated as follows:

• *Types of Studies:* Primary research that would ideally be considered might compare the accuracy of a designated bedside screening tool. Studies of certain types, such as editorials, commentaries, opinions, study protocols, and literature reviews, would likely be excluded.
• *Participants:* The studies would need to specify a particular setting, ensuring a thorough selection of diagnostic evaluations. For instance, it might be apt to include chronic patients aged 18 and above, across a spectrum of recovery settings.
• *Index Tests:* Investigations evaluating certain screening tests, tailored to detect or gauge the risk of a specific symptom, would be included.
• *Target Conditions:* The review could focus on studies that report on the accuracy of the screening tool in discerning the risk of a particular outcome.
• *Reference Standards:* It would be pertinent to include studies that contrast a particular screening tool, facilitating an evaluation against a professional benchmark.

To enhance the clarity and accessibility of the research findings, preference may be given to studies published in widely recognized languages within the field of medical research. Publications not directly aligning with the review's objectives or failing to meet predetermined quality standards may be excluded. Potential exclusions could involve studies with fragmented or inaccessible data. Additionally, preliminary investigations, publications lacking robust peer-review processes, or those predominantly relying on self-reported data without verification may also be excluded.

### Quality assessment of included studies

In the article selection process, utmost objectivity should be maintained. Two independent reviewers should individually assess each publication. This dual-review approach is designed to ensure every decision is grounded in a thorough evaluation, remaining free from individual biases. Should discrepancies arise during the selection, a third reviewer should be engaged to facilitate consensus, upholding the integrity of the selection procedure.

The risk of bias and methodological quality of included articles should be independently assessed through the qualitative criteria of Diagnostic Accuracy QUADAS-2 [9,10] and in accordance with the STARD Guidelines [11,12]. Systematic reviews of diagnostic accuracy studies frequently display notably heterogeneous results, stemming from variations in the design and conduct of the encompassed studies. Thus, a scrupulous assessment of the quality of included studies is imperative. QUADAS-2 should be chosen since it's recommended for use in systematic reviews of diagnostic accuracy by the Agency for Healthcare Research and Quality, the Cochrane Collaboration [13], and the U.K. National Institute for Health and Clinical Excellence. The tool should be tailored per review, adding or omitting signaling questions and crafting specific guidance on assessing each signaling question and using this data to determine the risk of bias. The STARD checklist should be adopted since it has been developed to enhance the completeness and transparency in reporting diagnostic accuracy studies. Authors should utilize the checklist to compose insightful study reports. Editors and peer-reviewers should utilize it to assess if the necessary information has been incorporated into submitted manuscripts.

**Fig. 2:**
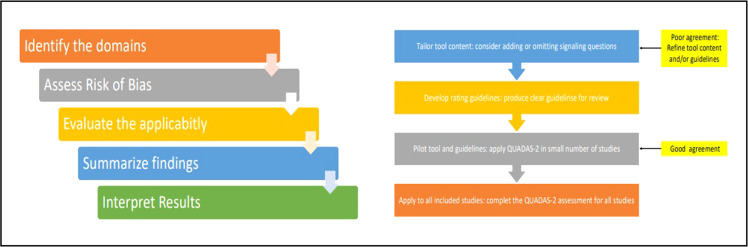
QUADAS-2 Assessment and tailoring.

These two evaluation tools should be pilot-tested to foster interpretative consensus within the team. Discrepancies should be resolved involving a third review author. The comprehensive algorithms of risk of bias and quality assessment (Fig. 2) should be available for reference via a link presented within the data availability statement (Supplementary File 1).

### Data extraction

The following main sets of data should be extracted from each included study:

• author(s)
• year and country of publication
• type of study
• population and setting
• healthcare professional performing the screening
• screening tools used
• diagnostic accuracy and reliability of screening tools used
• time for the screening to be performed.

In any case, it is advisable that two independent reviewers manage the data extraction to guarantee precision and thoroughness. Should any discrepancy arise in the extracted data, a consensus method should be employed, possibly engaging a third reviewer.

### Data synthesis

Typically, in a systematic review (SR), studies that are included get classified based on their primary and secondary aims. Defining the primary objectives is essential to hone in on the central theme (e.g., pinpointing dysphagia screening tools suitable for chronic post-stroke patients). Meanwhile, the secondary objective (e.g., gauging the diagnostic precision and reliability of these tools) acts to further refine the scientific emphasis. In a majority of these studies, the analyzed parameters of interest usually encompass sensitivity, specificity, and both positive and negative predictive values. Such data is organized into tabular forms and subsequently elaborated upon in a descriptive summary. The information extracted from these studies should be presented in accordance with its depiction in the original research. If substantial variability is detected across the studies, conducting a meta-analysis may not be appropriate. Should there be more than a couple of studies evaluating the same screening tool, their findings might necessitate amalgamation into a combined analysis of sensitivity and specificity. For example, in a reference review, the aggregated results would be showcased along with their respective 95 % confidence intervals.

### Implication for research

Rigorous evaluation tools, such as QUADAS-2 and the STARD checklist, combined with the dual-reviewer methodology, ensure that the selected diagnostic accuracy studies adhere to high standards of quality and relevance. Serving as a foundational approach to systematic reviews (SR) of diagnostic accuracy studies, research communities should be encouraged to adopt, adapt, and enhance this methodology in sync with emerging evidence and technological advancements.

### Limitations

A potential limitation of this review protocol may lie in its limited adaptability to different contexts, reducing its generalizability. Additionally, the rigidity of the protocol could lead to the exclusion of relevant research, and its application to diverse topics might introduce some subjectivity, influencing the coherence of the reviews. The complexity of diagnostic assessments and variability in methodological choices could also pose potential challenges in the protocol's application.

### Conclusion

This research protocol describes a standardized approach to systematically review methodology about a specific topic, such as for instance dysphagia screening tools. This methodology strives to be rigorous, transparent, and reproducible, emphasizing robust evaluation methods like the QUADAS-2 and STARD Checklist for diagnostic accuracy studies. It is hoped that this protocol can guide future researchers in such review studies, through a specific tool that supports the phases of the study process. The protocol is adaptable to many topics dealing with diagnostic accuracy, an element that is not always easy to interpret due to heterogeneity of tools and evaluation scales.

## Supplementary file

• Preferred Reporting Items for a Systematic Review and Meta-analysis of Diagnostic Test Accuracy Studies Guidelines (PRISMA) available at: https://doi-org.bibliosan.idm.oclc.org/10.1001/jama.2017.19163
• PIT Methodology available at: 10.1002/9781119756194.ch5
• QUADAS-2 available at: 10.7326/0003–4819–155–8–201110180–00009
• STARD Guidelines available at: 10.1136/bmjopen-2016–012799
• Supplementary File 1 (search strategy, algorithms of risk of bias and quality assessment of original study) available at: 10.6084/m9.figshare.24179532

## Funding statement

The authors received no funding for this research, neither from internal nor from external bodies.

## CRediT authorship contribution statement

**Marco Sguanci:** Conceptualization, Methodology, Writing – original draft, Writing – review & editing, Investigation, Visualization. **Stefano Mancin:** Conceptualization, Methodology, Writing – original draft, Writing – review & editing, Investigation, Visualization, Formal analysis. **Michela Piredda:** Methodology, Writing – original draft, Writing – review & editing, Visualization, Supervision. **Maria Grazia De Marinis:** Methodology, Writing – original draft, Writing – review & editing, Visualization, Supervision.

## Declaration of competing interest

The authors declare that they have no known competing financial interests or personal relationships that could have appeared to influence the work reported in this paper.

## Data Availability

Data will be made available on request.
